# Hospitals in Relation to the State, the Public, and the Medical Profession

**Published:** 1913-08-02

**Authors:** 


### HOSPITALS IN RELATION TO THE STATE, THE PUBLIC, AND THE MEDICAL PROFESSION.

The Section of Medical Sociology, at the eighty-first annual meeting of the British Medical Association, held at Brighton, under the chairmanship of Dr. R. J. Ryle, M.A., J.P., President of the Section, discussed on the Friday the question of Hospitals in their Relation to the State, the Public, and the Profession.

The President,

In introducing the subject and the speakers, said: That in our passage through life many of us might be described as persons who fell by the way, whatever might be the racial advantages or the supposed hereditary ones; many did not reach their goal in life owing to ill-health. His old teacher, Dr. Moxon, long deceased, said the function of the doctor was to protect the individual against the race. That might appear an obscure statement, but there was much meaning in the words. The function of the doctor was to extend the helping hand, and try to restore such, and hence the importance of the subject about to be discussed. The hospital might be said to be the point to which all the efforts put forth at this Congress conveyed. Except for minor ailments which were amenable to domiciliary treatment, the community was actually dependent on some form of hospital, using the term in its widest signification. From the time of St. Bartholomew's Hospital, London, 700 years ago, there had been gradually evolved the large number of imposing hospitals in the Metropolis and in most provincial towns, so that there was gradually coming about the ideal placed before the public in that wonderful book, "Utopia," by Sir Thomas Moore. At the present time most people preferred treatment in hospitals to being treated at home. Towards this two special factors must stand out in a host of discoveries, the antiseptic principle in surgery and the trained nurse. There were many people, organisations, and agencies which generously contributed to hospitals, but a large portion of the people knew little of hospitals, and appeared to care less.

### THE FUTURE ORGANISATION AND WORK OF HOSPITALS.

Professor Benjamin Moore, D.Sc., F.R.S. (Professor of Bio-Chemistry University of Liverpool),

In the course of an elaborate paper, said the subject of the future organisation and work of the hospitals as part of a national system for the combat of disease should be considered in a three-fold aspect: (1) In regard to prevention of disease; (2) as to the treatment of disease; (3) as to the increase of knowledge of disease. These problems were closely inter-related, and one of the greatest defects of the present mixed system of hospitals—partly under voluntary or charitable control, partly under municipal, and partly under Poor-Law control—was that all co-ordination of teaching and research, and of prevention and treatment became lost in a process of drifting. Hence all national character was lost from the hospitals, as also were the planning and execution of research into disease on some common scheme, the scientific diagnosis of disease in the effective way that sociology demanded, the treatment as a potent factor in prevention, and the education of the community towards disease as a primary sociological problem. In their place we had at present a patchwork of charity, and the doctoring was a palliative of the condition of the individual patient. The whole formed a system of administration, inadequate in some directions, and wasteful and overlapping in others, which reflected but little credit upon a country which was supposed to lead the civilised world in matters of hygiene. When the reformer turned his attention to hospitals he was met by the statement, made by well-meaning and charitable people, that the ethical training effected by charity and voluntary effort must not be weakened. Such an objector was a close ally of the municipal councillor who had hitherto attempted to bar the way towards the proper nutrition and hygienic supervision and systematic treatment of the school child, by the cry that it interfered with parental responsibility, which really meant an interference with the parental freedom to neglect the child. That, however, was now being beaten down, and a little more education of the populace was all that was necessary to start a similar movement to replace our present heterogeneous hospital systems by a properly co-ordinated system with local public control at each district, unified nationally by a public health authority. The beginnings of this movement were to be seen already in the applications being made to municipal authorities for assistance from rates in aid of powerful voluntary hospitals which had hitherto held their heads very high and disdained municipal or State assistance. This indication was also to be found in the grants from the State for the education of medical students to the London and provincial medical schools, which schools were founded on a purely voluntary basis, and had been supported by voluntary contributions. The National Insurance Act in the near future—partly by the additional strain thrown on the voluntary hospital system, partly by the involved reduction in charitable donations, and partially by the fact that State and municipal funds were now available, and patients were being taxed for non-voluntary contributions—would rapidly produce an increased rate of approach towards a national system of hospitals. Would this approach have the effect feared by its critics of destroying the benevolence and high character induced by the voluntary system? Would compassion for the suffering and friendless disappear because the hospitals would form the centre and basis for the modernised system of treating disease sociologically as an abateable scourge of the community? A brief examination of a somewhat parallel case might serve to indicate the rôle which voluntary gifts and charitable bequests might play in the future with regard to a national hospital scheme. The modern powerful municipal universities had arisen only within the past generation, yet some now had an annual budget of nearly £100,000; yet these were all financially dependent upon just such a happy combination of voluntary aid and municipal rates, State taxes, and payments of fees as might be instituted in support of municipal or State hospitals. When it was proposed for the large provincial towns there were some wise shakings of the head, just as there were now over the idea of the municipal or State control of hospitals; the same doleful forebodings as to the drying up of private charity. But, happily, all the gloomy forebodings had been falsified by the march of events, and the municipal and provincial universities had grown in numbers and in strength, and were now making themselves felt as a powerful national stimulus by the modern and democratic spirit which they had introduced into higher education. What the modern universities had done for education, a modernised hospital system was capable of doing for the study, prevention, and treatment of disease, and its financial support would be as certain as that of the universities, and derived from the same types of resources : State, municipality, and private donor would combine to aid one another in the evolution of the best; and their united best would be better than that of any one of those great forces acting alone, or all acting separately as at present. In the Parliamentary Bill now being promoted by the City of Liverpool powers were being taken to make grants to the funds of two voluntary hospitals, one of which was the premier general hospital in that city. In the same city, at about the same date, an appeal was made to the charitable by a newspaper for £2,000 for research and treatment in the same institution, and within three or four days the amount had been subscribed. Here was the sort of combination which he advocated. Medical students were being taught their profession in that institution, and on their account the university received State aid through the Treasury grant, and that was utilised to support the clinical school, from which the hospital in turn received much aid in service. The second great bugbear in the minds of those who were opposed to municipal or State hospitals was public control, as opposed to the disinterested control of the finest and most lofty-minded citizens, actuated by the noblest motives of religion and philanthropy. That was also feared in the case of the universities, and it had been shown to be quite groundless. The order of sociological development was the usual one in such enterprises, but the veterans of the voluntary movement feared the new control which State support might involve, but they would be surprised at how little it would change the spirit of things, and at seeing hereafter the same men sitting on the boards and committees, only sent by other electorates in other capacities. There need be no dislocation, no removal of old and faithful workers of the voluntary movement. It was sound sociology to say that where there was municipal or State financial aid there must be a corresponding measure of control of policy by these public bodies, but in all communities the personnel of the active-minded citizen was the same on voluntary bodies and on municipal ones, and there was a linkage of those most willing and fitted to serve on the committees. It would probably be admitted on all hands that, valuable as had been the work of the voluntary hospitals in the past, and valuable as had been the example they had held up for the municipal and Poor-Law hospitals which had taken up the large balance of work that the voluntary institutions could not touch on account of lack of funds, yet the voluntary system, acting alone, could not solve the sociological problem of the treatment and prevention of disease among the whole mass of the industrial population. The pity was that all its ennobling influence should be shed upon one section of the hospital system, while the other and numerically greater portions were left untouched, often to sink to a lower level, which invited an invidious comparison. The need was unification, and the shedding of this benign influence through the whole mass. This position, already clear to the thoughtful sociologist, would become intensified and apparent to all at that early date when statesmen, realising the nature of the problem, would extend the operations of the national scheme against sickness to the women and children of the community. And some mechanism would have to be provided for putting each practising physician in intimate touch with some hospital, and providing him, for his work's sake, as well as for that of the health of the patients, with ready access to its wards. The expense of this would be too great to be provided by the donations of the wealthy and voluntary collections from the poor. Then voluntary contributions could be devoted to increased facilities for research, and the further advance of knowledge; private donors could found Research Fellowships and Chairs, and fit up expensive $x$-ray and radium installations, and subsidise investigations of particular diseases. It was an absurdity to argue that human nature would change or deteriorate because the State as a whole recognised the performance of its duties towards a national problem, which was perhaps the greatest in the realm of sociology. Charity, as we now understand hospital charity, must be rigorously excluded; the old idea of charity must be replaced by the scientific realisation of the hospital as the pulsating life-centre of a little corps charged with the maintenance of the highest degree of health in a given district, and correlated to similar corps and units all over the country. The medical profession should look upon the hospital not as an institution for their leisurely treatment of the sick, but something with which in the entirety it was organically knit up. The unit would seem to be 80 to 100 beds for each 100,000 of population; and it should have 60 to 65 medical men attached to it, giving whole-time service in various capacities. The work of the medical officers should be varied from time to time, so that each might acquire a full knowledge of the work, some being set apart for visiting in the district, and being closely connected with the hospitals as headquarters. Such a system could be demonstrated to be both more economical and more effective in its purpose than our present haphazard system of running medicine as a business concerned almost solely with the task of patching up the individual patient after disease had already made its inroads.

### THE FINANCES OF THE GERMAN HOSPITALS.

Dr. J. G. Gibbon, D.Sc.,

Dealt with the finances of the German hospitals. He said municipal hospitals were plentiful in Germany. In the municipal areas hospitals were provided by the municipalities, and the State helped in various ways. In Prussia the State built and maintained the university hospitals, but did not give assistance to the local hospitals, but in Würtemberg the State helped the local authorities in the provision of hospitals. A large number of sanatoria had been erected by the Insurance authorities, and there had been a great increase in hospitals in recent years, largely on account of urban communities having increased, and the restrained conditions of life were not favourable to recovery from illness or to home nursing. There was still considerable pressure on hospital accommodation in Germany, and several municipalities had found it necessary to extend their hospital accommodation. The ultimate responsible authority was the council, under which there would be a committee, and the actual administration was in the hands of the director. The doctors considered there should be a medical director for the whole institution, but that was not the system which universally prevailed. In some cases there was a medical director for the medical work, and a lay director for the other, a situation which did not please the doctors. The large hospitals were divided into separate departments, each with its medical officer and assistants. The principal medical officer was allowed to have consultative work from outside, but was not permitted to participate in general practice. In the small hospital it was essential that the doctor should be some one who had general practice as well. There had been an alarming rise in the capital cost in Germany in recent years. In ten out of eighteen hospitals which had been built during the last decade, the cost, excluding that of the land and equipment, was under £200 per bed, but in five it was over £300, and in one it was over £450. The size of a hospital should be, according to some authorities, 100 to 150 beds, but many German hospitals exceeded that; Hamburg had one with 2,000 beds. Prices of material had risen considerably, and the advances in medical science made necessary more expensive apparatus. In addition, too little homage had been paid to economy, and there was a rivalry in different towns for the possession of handsome buildings. There were now signs of a reaction against this, and the ratepayer was claiming more consideration *before* he entered the hospital as a patient. It must be remembered that the conditions in various parts of Germany varied enormously, more than was the case in this country. The cost of treatment and maintenance, excluding capital expenditure, varied from 2s. 6d. per patient per day to 6s. and 7s. The usual cost was from 3s. to 5s. This was largely due to the differences in the treatment and in the proportion of beds occupied, as also to the number of very acute cases. Also there were different classes in hospitals, the cost for which varied, and prices varied in different districts. Salaries were a considerable item; with the cost of instruments and drugs it might be one-third to two-fifths of the total expenditure. In fifteen hospitals the staff per thousand sickness days varied from eighty in one place to over 200 in another. There was also a considerable difference in the number of medical practitioners employed. They numbered from five to sixteen, eight to twelve being the general run. One authority said that where there were thirty beds there should be at the least one assistant, and that was essential in the case of fifty beds. The average salary per practitioner employed, including the assistants but not including board and lodging, came to £150 to £200. The medical superintendents might get £125 to £600. Some might be provided with a dwelling, some with a pension. The ordinary assistant would receive £50 to £100 a year, with board and lodging. A resolution had been passed by the "Verband" that assistants should receive not less than £60 with board and lodging. Assistants might receive some slight additional remuneration from the certificates. In Germany it was common to take nurses from associations, not to employ them direct, as in this country; in some districts religious Orders were applied to for nurses, the latter working for a much lower remuneration than the others, a considerable amount of stress being laid on her calling, remuneration being apparently regarded as secondary. But grace rarely made up for lack of training. In some cases no payments were made by hospitals to the nurses themselves, but simply to the associations supplying them. With regard to the sources of income, that derived from endowments was not large; they were chiefly from the payments of patients and from the long-suffering public exchequer. The superior class paid more, and had different food. Their scale was from 6s. to 12s. a day; the second class 4s. to 7s. a day, and for the third class 2s. to 3s. a day. The food of the first class, generally speaking, cost three times as much as that of the lowest class. It might be said, roughly, that the patients in the first class paid more than the cost of treatment and maintenance, those in the second class paid about the right amount, while those in the third class paid less than the cost. Some additional payment might have to be made for some special treatment. Of course, most of the patients belonged to the lowest class, and they were the ones paid for by the insurance societies, and to these belonged the Poor-Law patients. Some societies said the doctors sent cases too freely to hospital, and the doctors retorted that the societies used the hospitals too freely; and a demand had been made that the assistants in the hospitals should make common cause with their fellow-practitioners outside. A large proportion of the patients were treated at the expense of the Poor Law, so that a considerable amount of money had to be met out of the public exchequer. In a group of fourteen hospitals, in four the average amount which could be met from the exchequer was at least 3s. 6d. per sickness day. In three it was 2s. per sickness day, and in only two was it under 1s. The charges on the local or national exchequer for hospitals were very heavy, and an examination of the subject did not enable him to regard the position of the German hospitals as altogether a satisfactory one, nor were the conditions in that country necessarily applicable to a country like Britain; it was necessary to consider individualities and local tendencies. The German experiences should not, therefore, be taken and applied automatically to this country.

The President

Said he did not think any apology was needed for bringing into the Section's work a discussion on German hospitals, because that country had set an example to the civilised world in the matters of thoroughness and organisation wherever there was a relationship between the State and the community. He called upon Professor Pfeiffer, of the Municipal Hospital, Essen.

### THE RELATION OF THE GERMAN HOSPITAL TO THE PROFESSION.

Professor Wilhelm Pfeiffer, Medical Superintendent of the Municipal Hospital in Essen,

Read a paper on the German Hospital in its Relation to the Medical Profession. He said it was essential that the medical superintendent should be the highest authority in the hospital on general as well as medical questions, and he should have an efficient staff, both lay and medical, and under no circumstances should the doctor be subordinated to a lay official. The medical superintendent should receive a fixed sum, in proportion to what he had to do, and he should be entitled to a pension. Also he should, when practicable, receive medical fees of patients over and above the hospital dues. The hospitals of Germany he divided into three groups: (1) the State hospitals, which included the university and provincial and district hospitals; (2) municipal hospitals; (3) private hospitals, included in the latter being the so-called "Confessional Hospitals," which belonged to religious Orders or had been established by grants or bequests. The medical superintendent of the State hospitals, when incapacitated by serious illness, was excused from giving lectures without reduction in salary. The salary in the State hospitals varies from £250 to £500 per annum. The assistant doctor receives between £60 and £240 per annum. The large municipal hospitals have different departments for the various specialties. In most of the cases the administration of these hospitals is in the hands of a lay official. In many cases he ranks with the medical superintendent. The medical superintendent has the right of deciding all matters of a purely medical character. Twenty per cent. of the appointments carry with them the right of a pension. The salary fluctuates between £250 and £500. In the State hospitals there are assistants whose salaries vary between £90 and £330 per annum. There is usually an assistant for every ninety beds. The conditions are worse in the private hospitals, where the salary ranges from £90 to £250 per annum. The medical superintendent in large hospitals is entitled to have an hour set apart for consulting work not connected with the hospital. The relations of the general practitioners to the hospitals are in most cases limited by the fact that they send to the hospitals only those patients whose treatment can be more conveniently undertaken in the hospital than at home. When the patient leaves the hospital the treatment by the hospital is finished.

### GERMAN NATIONAL INSURANCE AND HOSPITAL PROVISION.

Professor Grober (Jena),

In the course of a very careful paper, said his discussion of the subject would meet with some difficulties, because the organisation of the German hospital was totally different from the English, and secondly because England was just beginning to produce legislative insurance. In Germany, working people earning a certain amount were obliged to be insured against acute illness of six to twelve months' duration, and people were insuring against illness of a permanent character. There was a social insurance, especially for the increased nursing and treatment in hospitals. Formerly, people who were only slightly ill would not think of going to hospital, but now they were anxious to do so, especially as there was a lack of proper facilities at home. The social insurance against accidents also required treatment, and the hospital could grant them more constant medical surveillance and better control of their hygienic conduct than was possible under home treatment. The number of hospitals in the country had increased from 888 to 2,314, 300 per cent. increase; while the number of beds had risen from 37,000 to 160,000, or a rise of 400 per cent.; and the number of patients from 211,000 to 1,304,641, or nearly 700 per cent. In 1877 the number of beds provided per 10,000 inhabitants was 14.2; while in 1910 it was 19.5. In the big industrial German towns there was still a complaint of lack of beds. Insurance legislation in Germany was now increasing. The German law for public hospitals was different from that for private ones. The State of Prussia owned the university hospitals, also the provincial lunatic asylums and provincial schools. The development in German industry and trades had enabled the towns to take up the care of the suffering in a more complete manner than ever before. Some private hospitals belonged to doctors or nurses or companies. Before a private asylum was allowed, the owner was examined concerning his capability to manage a hospital, and they must conform to rules framed for their control. The conditions had to be strictly maintained. Monopoly did not exist. The chief duty of hospitals was to cure sick people; to treat and nurse those who could not recover was only of secondary importance. Many celebrated hospitals were formerly only infirmaries for the poor. Town hospitals were often obliged to perform other functions, such as taking in persons found unconscious in the street, also those who were suspected to have hurt themselves, and had to be watched. In addition, the hospital was expected to educate the inhabitants as to how to lead healthy lives and how to avoid prison and bad health. The cost of furnishing and maintaining hospitals had risen greatly during the last thirty years, chiefly due to increased cost of land, clinical fittings, lighting, sanitary arrangements, and higher wages. In some hospitals the aim had been to build hospitals pleasing to the eye, which meant greatly increased cost, sometimes to twice the former figure. The greater number of patients now needed more rooms than formerly, and so the technical arrangements were now more complicated. *X*-ray installations and finer instruments had definitely increased the cost; and the same might be said of specially nourishing foods. Native patients requiring treatment at hospital had to pay less than strangers. The prices at various hospitals in a locality were made uniform, so as to obliterate elements of competition. The cost to a third-class patient was two to four marks daily, and for one in the first or second class, six to twelve marks. Often patients requiring specially expensive apparatus were charged extra. Ordinary charitable gifts in the case of some hospitals did not exist. In only one case he knew of were the charitable gifts at any definite and regular height. Still, bequests to hospitals were not uncommon. Since 1911 a method of economy which had been practised was separating the cases of chronic illness and convalescence, which did not need the same elaborate treatment as the more serious cases. The former were therefore sent into country establishments situated near the towns in question. The State had reserved to itself the right to inspect the hospitals, and, generally speaking, the Prussian example had been followed. Every hospital must have rooms for the examination of suspicious cases the character of which was not at once evident. Every ward must have a day-room and regulations for heating, water supply, etc., bathrooms, and lavatory accommodation. The bigger hospitals must have two special buildings, one for kitchen and housekeeping matters, the other for disinfection, and a mortuary. The rules had kept pace with the progress of medicine and hygiene during the last ten years. The number of clerks and officers was now greater than ever, and in some hospitals there was a danger of household matters being considered equal to the claims of medicine, and in those instances the position of the doctor was an unpleasant one, especially as the doctor was, by the force of public opinion, made responsible for everything in the hospital. The nursing requirements were not completely met, because the inclination on the part of German women to become nurses had diminished. One nurse to every ten beds was the usual number reckoned on, but in some cases one nurse had to look after twice as many. With some nurses the chief motive was a religious one, remuneration being regarded as secondary in importance. There were nursing schools acknowledged by the State. The salaries of nurses varied enormously, from 80 marks, or £4 per annum, with board and lodging, to ten times that sum. It was intended to equalise these conditions more. In Germany the reception of a patient into a hospital was not considered an act of charity, but was in the nature of a contract between the owner of the hospital and the patient who paid and the institution of which the patient was a member. Owing to the needless luxury in some hospitals, the requirements of the patients had been greatly increased, and the remedy for that was a more interested control on the part of the public; the interior working and mode of living in the hospitals should be known to every fairly educated person. The hospital would prove to be the great improver of public health, in which the Emperor and other sovereign families took a deep interest. The value of the German hospital had not yet come to an end. Possibly it was still too beaurocratic, but it was getting on very well. It would be better if hospitals were left more to the care and supervision of doctors specially gifted and trained for that purpose. There were some good examples of that in the United States and Austria, but not generally in Germany. He hoped the experience of hospitals in Germany during the past thirty years would help those responsible for British hospitals to go on in the most useful way, first to the individual patient, secondly in the matter of the public health, and thirdly in their relations with the British Medical Association.

### A REVIEW OF THE BRITISH HOSPITALS' OUTLOOK.

Sir Henry Burdett, K.C.B., K.C.V.O.:

Ladies and gentlemen, the last speaker said that the German hospitals have not yet attained to the full zenith of their efficiency or of the good which they hope to accomplish, and will one day be able to accomplish. Certainly that is true of the British system of hospitals. And we have never in the whole of my experience of hospitals, which extends over forty-eight consecutive years, had before us an outlook so serious and so essentially calling for the best brains, and the most careful consideration of every statesman and every member of the medical profession, with all others who have had to do with hospitals, as we have at the present time. (Hear, hear.) Thanks to the voluntary principle, we have a system of hospitals which, I confidently declare, after having inspected them within the last few years, and having also inspected the German and other foreign hospitals, to be, on the whole, the best in the world. That is not simply a statement given by an Englishman because he is proud of his institutions, but is given by a man who has devoted his life to hospitals, and it is a deliberate opinion. (Applause.) With reference to German hospitals, which I spent time last year in inspecting, you have heard these interesting papers, and I am sure we are grateful to the gentlemen who have read them. Some of them have said that they do not think we can learn from the German hospitals. But I am sure we can. It is perfectly wonderful, indeed an education, to compare the German hospitals twenty years ago with what the best of them have become to-day. There are among recent municipal hospitals in Germany institutions which the Germans are apt to consider cost too much money, but which, I maintain, are a glory to Germany and a help to the whole hospital world, because the men who have gone into the question of such hospitals have had a free hand, and have produced some excellent results. Now, in this country, what we want at this stage, as a first essential to the wise solution of our hospital problem, is co-operation and intercommunication between all voluntary government and municipal institutions for the relief of the sick. We must get that. We have first to recognise what we already possess, then to build upon this foundation, and out of our existing agencies to evolve a perfect health service and a perfect hospital system. The need for such a policy has been emphasised by national insurance. National insurance, which under the Act has come to stay, could not to-day exist as a financial proposition apart from the voluntary hospitals.

### Position of Hospitals under the Act.

If it were not for the voluntary hospitals, and for the services which the voluntary hospitals are rendering to national insurance to-day, Mr. Lloyd George's Act would speedily come to an end. That is an actuarial fact which every actuary in the country knows, and which every business man recognises too. (Applause.) Yet the present position *qua* hospitals is most unsatisfactory. The only recognition in the Act of hospitals is under two clauses, 12 and 21, which might lead you to suppose that, as the very lifeblood of national insurance is hospital provision for in-patients—I shall not deal with out-patients to-day—and national insurance could not go on but for the voluntary hospitals, these clauses would help to provide the money required. And yet unfortunately these two clauses, 12 and 21, in the financial sense, are not worth a snap of the fingers. Clause 12 provides that by agreement hospitals may, in the case of a patient being an insured person who has no dependants—imagine them having no dependants if there was money in it! (laughter)—first make an agreement with the society, and then the hospital may get something towards the cost of maintaining these insured persons. It would in the best of circumstances be difficult enough to recover the money as it is; but you must remember that the hospital has to come to an agreement with an approved society, or with one of the insurance companies which are working national insurance in this country. That makes it almost an impossibility for the hospital to get sixpence. Why is that? Because these approved societies and insurance companies are in competition, and as a result their policy is to withhold the money belonging to the person without dependants until he comes out of the hospital, when it will be spent for his benefit in a way most advantageous to him. That is a non-committal policy; it is a business policy, and to the people who are competing I have no doubt it is useful. But it is hopeless with regard to the hospitals, or in regard to any financial benefit coming to them.

Let us look at Clause 21, which provides that these approved societies and insurance companies may give hospitals subscriptions and donations. They "may." (Laughter.) I have been working for hospitals, getting money for them, for nearly half a century, and I do not know any experience or any occasion when I ever got anything in the way of money from this source which was good value, for the reason that in the case, say, of a society of 100 members, subscribing five guineas a year they consider they are very handsome in their gift, but they wish to make it a condition that you shall provide hospital care for any of the members. Therefore, that clause with regard to the hospitals is of small value, if any. So we are driven back to this: that the voluntary hospitals are in the position which they have nobly occupied and do occupy to-day. It is the position of giving all this medical aid to in-patients at the enormous cost of hundreds of thousands a year which is given in our country to support this National Insurance Act, and the hospitals have not got anything in return for it. It is very important you should have this position very clearly in your minds; it is the bedrock from which we in England should take our new departure, and so the voluntary system would become stronger than ever.

### The Bogey of State Control.

You cannot hold over the voluntary hospitals the bogey of Government interference and control where the Government are the debtors. The Government will have to provide all the money the hospitals cost to maintain before they can claim to control the hospitals; but they will never provide this money, and they cannot therefore claim to control the hospitals. We may do what is done in Germany—i.e., the Government might send inspectors over the hospitals and inspect the wards where the insured are treated and paid for on a *pro rata* basis; but there can be no question of taking charge of the management or control of the voluntary hospitals, for the State is very much in debt to voluntary hospitals! Politicians are the curse of the sick to-day in this country. (Applause.) I have fought the battle of the sick, and what do the hospitals get? Nothing. The cause of the sick is nothing to the politician unless you can serve Lazarus up in some form which is going to be attractive to the electors. We are not politicians here to-day, and I am not talking politics. Hospital workers and managers are people who believe in the Parable of the Good Samaritan, who have given the best of their lives and energies to try to make the lot of the sick better than it has ever been before; and we are strong enough to protect and defend the institutions upon which the sick have to depend in this country.

I have said that we are asked, as hospitals, to fulfil a promise made by politicians. What was that promise? That national insurance could give adequate medical treatment. Adequate medical treatment has had to be provided for the insured by the hospitals, as I have shown, and it is essential that in-patient treatment should be provided, and provided at once, because the very backbone of our existence as a nation is that the bread-winner, when struck down by accident or overtaken by disease, must be at once put under those conditions of medical treatment and care which will most speedily restore him to health and enable him to go on maintaining his family in comfort and prosperity. The present position of our voluntary hospitals is therefore that the State is under an enormous obligation to them. What is the future position? It is that we have to remember that we not only shall have to provide for the bread winners, who are the insured persons to-day, but that politicians are already saying they are going to provide insurance for the families and dependents of the insured, as in Germany. What will be the effect of that? An enormous pressure upon our hospital beds. It is true that in the first six months, or whatever period it is, that the Act has been working these insured persons have been taken in, and, on the whole, the hospitals are going on well. But they are not going on so well as would appear from the silence or the quietness at present, and for this reason, that the waiting lists of in-patients at hospitals are growing and extending, and they have very heavy lists of patients who ought to have in-patient care, and in some of the larger hospitals already there are as many as 250 people waiting. And that state of things will get much worse when we come to the extension of insurance to the families.

### Pay Wards and Pavilions.

There is another thing that they have faced in Germany, and that is that the time has come when all classes of the community, having regard to the development of medical and surgical science, are properly coming to demand that they shall have hospital care for them, and that it shall be on a basis which will enable them to pay for it, when they require it, at a fair rate. And the only way in which that can be done—and I am speaking as one who started the first pay hospital in this country in 1880, which has been running successfully ever since, has been rebuilt in the period and is to-day as prosperous as ever—I say the only way in which this can be done for the general body of the people is by pay wards and pay pavilions attached to our voluntary hospitals wherever it may be possible. And you must have that for two reasons: first of all, it is the only way in which you can provide the adequate means required for treatment; and, secondly, it is the only way in which you can provide it without objection by the medical profession. The rights of the medical profession can alone be protected under the pay pavilions system. There is in London an opening when St. George's and Westminster Hospitals are amalgamating, which will give a great opportunity to supply a modern, up-to-date hospital, so far as construction is concerned; it should not only comprise a big general hospital for free patients, with a great medical school fully equipped with laboratories and all the departments required, but it can also give us pay pavilions on a proper system, as a pioneer of the sort of thing we want. This suggestion is not very new, for I would remind you that in 1884, after an agitation for pay wards in this country, the London Hospital had a special Act of Parliament passed; and in that Act they took power to establish paying wards at Whitechapel and to treat in them paying patients to the extent of 10 per cent. of the in-patients admitted to treatment.

There is in England an enormous need for immediate preparation. We have to prepare for an increase in the number of the insured, for the demand for pay wards, and for the tightening up of our system of hospitals and health services by co-operation. And the first consideration will be the cost as an essential factor. It is said that the alternative is a system of State hospitals. The Poor-Law Commission went into this question, and found that, on the lowest estimate, the effect of having State hospitals in England must be that it would add at once twenty millions a year to the National Budget. Is that a proposition which any politician dare put forward? I do not believe it. In view of the present state of taxation and the amount of our national expenditure, we need not trouble about a national system of State hospitals. The cost would be prohibitive. Another reason is that I am certain British people will never give up their position, their birthright, of being treated in hospitals as individuals where their feelings and sentiments are the first consideration, in exchange for a system which regards them first of all as material for science. Therefore on these grounds I do not trouble myself greatly on this question of State hospitals.

### How to Provide the necessary Extensions.

Every voluntary hospital should look at its present circumstances. Many of them have got unused sites attached to them, and they ought to consider all their site and what it is capable of providing in the way of additional buildings, pay wards, extensions, or whatever may be necessary in these respects. There are many hospitals of this kind. One I went over the other day at Tottenham, where they have a population of half a million people, and they want 500 beds. There are only 125 beds, but there is ample room for building. If they did that, it could be rapidly and economically done, and it is a practical suggestion. Others could obtain additions to their sites at a reasonable cost, and they should consider what additional site they could have if they required it. It is the extension of our system which is needed and the maintenance for the additional beds for paying patients and insured patients. What cannot be raised by subscriptions is the cost of buildings and extensions. This must be met by loans, and not by appeals to the public; and those loans can, I have ascertained, be granted by using the credit of the country on a basis which is recognised and adopted by Parliament in regard to municipal enterprises. Remembering what I have said about the position of the Government or Parliament and the debt they owe to the voluntary hospitals, I am glad to find it is recognised that when the time comes for new buildings there will not be any difficulty about the money to pay for them. I cannot to-day enter into the details of this, but it is a practical proposition, as a matter of business, that I am prepared to explain to anybody who writes to me—I do not touch it in more detail to-day, owing to lack of time. But you can take it from me that it is a practical proposition: the difficulty as to buildings will be solved. The difficulty as to maintenance will be met, because it is certain to me that the in-patient treatment of insured persons must ultimately be paid for *pro rata*. Approved societies will have to pay for the cost of maintaining their cases in the voluntary hospitals, or wherever they may be treated. The matter is one of business between the State and the hospitals.

### The Future of Poor-Law Infirmaries.

We shall want more beds than we have in the voluntary hospitals; and we have in this country some good Poor-Law infirmaries and a very fine system of hospitals under the Metropolitan Asylums Board. Those two groups of establishments are injured by the fact that they have "pauper" written across them. But the time has gone by for that taint longer to be attached to these buildings and institutions. They ought to be made public hospitals, and they should be made as available for any person to go into without any taint whatever as the voluntary hospitals themselves. Such a change has been recognised in Government quarters, and I hope it will speedily come, that they will be made into public hospitals. If you remove the pauper taint, make them public hospitals, and open them for clinical purposes, they can become fully equipped with modern appliances at very moderate cost, and should have an adequate nursing staff. I should like to see the whole of the health (fever) hospitals brought into co-operation everywhere. That is the one blot of this country—the unpreparedness to be found, especially in smaller fever hospitals. The existing hospital accommodation, by co-operation and preparation, would thus be made equal to the needs of the population at the smallest possible cost. It is largely a matter of reorganisation and making available all the means already existing to brace up the whole of these agencies and make everything ready for the work which will soon have to be done owing largely to the introduction of National Insurance.

I should like to see these administrative changes with a department for the voluntary hospitals under the control of representatives of the voluntary hospitals throughout the country; and a department for public hospitals under the Government. The proposed plan is economical, statesmanlike, efficient, and would be popular. I should like to say here that I consider—although I know the decision which has been come to by the British Medical Association with regard to the payment of doctors who treat patients in hospitals—that that decision will not continue long. It only applies to the condition as it is to-day; it cannot continue to apply to the enlarged system of hospitals and the perfected health service which this country needs and must shortly have. I want to see, at any rate, justice done to the younger men; I want to see the medical profession, like other professions, put a premium upon the best brains. Every young man should, when he has taken a high degree by virtue of his brains, be helped to qualify for a high position in the profession and be liberally paid as medical and surgical registrar and in other junior appointments, so that he may be enabled to go on with his work, and to make his free choice as to whether he will wait for the best or whether he shall enter general practice. I want it to be so guaranteed that we shall have the best brains in the highest posts.

### The State Medical Service Agitation.

There is one other thing I want to say. There are rumours, and there have been circulars and announcements, and I have seen a proof of a paper, all intimating to us that we are to have a State Medical Service agitation. I have looked into this question for a good many years, and I have studied it a good deal, and I may tell you at once that this cock will not fight. (Laughter.) That is the simplest way in which I can put it to you. For the cost would probably be forty to fifty millions a year. It would degrade medical practitioners by reducing them to the position of a salaried class, with strictly limited incomes, and it would remove the safeguards which at present constitute the birthright of the British race to be treated in hospitals as individuals needing sympathy and tenderness, and not as mere material for science.

### The Tragedy of Sanatoria.

One other thing I want to say is, that sanatorium benefit ought never to have been included in the National Insurance Act, and it would not have been if it had not seemed apparently such a wonderful asset for the politicians. It will have to be totally reorganised. At the present time I am continuously having inquiries to know what can be done for these poor people who want sanatorium benefit and cannot get it. It is not the fault of Mr. Burns, of the Local Government Board, because he has provided 8,000 beds for these cases; but it is a fact that sanatorium treatment is not a cure, as has been represented by politicians. It is merely a palliative, and I am strongly of opinion that, like Palmerston's forts, if you were to cover the country with sanatoria to-morrow, in twenty years they would not be wanted for the purpose, and they would remain as monuments of what to avoid in legislation.

---

### The General Discussion.

Professor J. A. Lindsay (Belfast) Considered that no more important work had come before the Association than this question of hospital administration, and he felt deeply grateful to Professor Moore and the speakers who followed him for their illuminating contributions to the subject. It was well to take note of what their German friends had said about their own hospitals, not necessarily for the purpose of imitation, but as a source of suggestion. When speaking with a lady in Brighton on the subject, she remarked: "The Germans are so horribly efficient." He would not favour the importation of the German system of hospitals in all its details into this country, especially the method of having three classes of patients, and varying kinds of foods for those classes. Also the German scale of salaries left something to be desired. The idea of having pavilions he thought had become almost obsolete. The lesson Germany taught us was that of the importance of organisation; however well many things in our country might work, our organisation was none too good. Our hospital system had grown up to a considerable extent in a haphazard way; we had never thought out the question of the function of the hospital in relation to the city and to State. Owing to the lack of correlation there was much overlapping and unnecessary expense. There was, with us, no direct responsibility with regard to the treatment of sick persons whose social level was above that of the pauper. The latter were treated well and freely, but for the better classes there were only private and very expensive hospitals. The large middle class was most inefficiently provided for in this respect. (Applause.) He would like to hear discussed the question of the relationship of the medical practitioner to the hospitals of the future, for the hospitals would be doing a larger and larger work. So far, the general practitioner had not had enough say as to the cases admitted to hospital; in many of our cities that was a source of dissatisfaction, and he hoped to see it solved. He did not think hospitals should be built and maintained on a luxurious scale. The King's Consumption Hospital at Midhurst cost about £700 per bed to build, which seemed to be about thrice what it should have been. He concluded by declaring that the question required a good deal of hard thinking, as we were now on the verge of a new epoch.

Dr. Charles Drysdale

Desired to draw attention to what the community as a whole gained by hospitals, and he would do so by relating what he saw during a visit to a brand-new system of State hospitals and asylums in Hungary. That country seemed to be the only one which had introduced a State system of asylums and hospitals for the care of infants and their mothers. The institution he referred to was a magnificent one, and it had been in operation ten years. They had seventeen asylums distributed over the towns, and they looked after 55,000 children. The facilities for proper care and upbringing were splendid. If the mothers were working women, they were given assistance so that they could stay at home for a time and nurse their children in a natural manner. The infantile mortality in Hungary in those institutions had fallen to fifty per 1,000, although the children cared for there were the most weakly, and it compared well with the 200 per 1,000 mortality for Hungary as a whole. He went as a sceptic, and he had to confess that he was not converted, as he was not likely to be until he had found out the effect on the community as a whole. Since his return he had inquired into the effects on the community as a whole, with the result that he remained a sceptic. He showed the figures in diagrammatic form. Until a few years ago the mortality was coming down rapidly, but since the establishment of the child asylums, that mortality decrease had been checked and was no longer seen. He belonged to a school of economists who fancied that when evil was chased out of one place it was apt to be driven into another unless the fundamental phenomenon were inquired into. The general mortality had also ceased to come down since 1901. He did not think the hospitals or the medical profession had reduced the death-rate. If his hearers were to make a comparative study of the vital statistics they would be driven by inexorable logic to the same conclusion. The hospitals dealt with the very poorest and most diseased; and, while their sufferings were alleviated, why should not the profession and the country do the right thing—namely, suggest that these defective people should not confer their defects upon the succeeding generation? Without such measures the efforts for the general improvement of the community would be in vain, but with them there must be a great improvement in the race in five or six years.

Dr. Gervis

Thought few of those present would agree to abolish all hospitals and trust to remedying the evils of life by lessening the birth-rate. The consensus of opinion seemed to be that we require more hospitals, and of a greatly improved sort. He would like to speak from the standpoint of the general practitioner. Why did the medical profession require improved hospitals? Medicine was becoming such a vast science that it was impossible for any one man to make himself master of more than a small portion of it; and hence the principle of co-operation in regard to the sick was introduced more and more. The more deeply the subject of disease was gone into the more it was found that, although specialism must develop, the interaction of the various organs on each other was so intimate that the services of various specialists were required for adequate treatment. That was workable in the case of the well-to-do patient, because the medical man in charge could call in various specialists in consultation. The poor man had the same advantage in the hospital. The public should realise that the great advantage of a hospital did not consist solely in the carrying out of operations. Listerism had shown how necessary was that ritual for success in treatment, and that was obtainable to the full in the modern hospital; and the hospital was also most useful in the matter of diagnosis. The hospital of the future would be the centre for the medical work of the whole town; and the medical man whose patient was in the hospital should be able to attend him there, for patients liked their own doctor to attend them. He believed there would not be any real difficulty in obtaining the necessary money from the State in conjunction with the charitably disposed members of the public. Sir Henry Burdett had said that the sick person was nothing to the politician; but the voter was everything to him, and if the working man sufficiently realised the importance of the hospital the needed money would be forthcoming at the next election.

Mr. Joseph Fels (Philadelphia)

Said he was not a doctor, but was engaged in trying to get at the bottom of things. He agreed with the remark concerning the use of spare land for hospital sites, and pointed out that in London there were 14,000 acres of unused land at the present time which was held up by the landlord speculators. The question under discussion was an economical one. In Germany there were 134 cities which, in their municipal character, took land values in taxation for public purposes, yet not one city in Great Britain did the same thing. If the housing of the very poor were better attended to, there would be less need for hospitals. There should not be such dependence on charity as there was in Britain to-day. If 1 per cent. on the saleable value of unimproved land were levied as a tax, some hospitals would be rendered unnecessary, and others could be improved. There should not be such a great amount of illness in any country as existed at the present day.

Professor MOORE,

In reply, said it was clear from what the German speakers had said that if England were to nationalise the hospitals she would be the first country in the world to do it. The voluntary hospitals of this country had done only one-fourth of the medical work of the country, though they had done it well. One very important thing to be on the watch against was the production of an inferior medical profession by descending to excessively low salaries; men must be paid well for doing the work which was worthy of the best intellect of the country.

Dr. GIBBON,

In reply, pointed out that the standard of nursing in Germany could not compare with that in England, for in Germany the training occupied only a year or eighteen months.

Dr. PFEIFFER

Replied that most of the patients in the German hospitals were of the third class. In Essen only 5 per cent. to 7 per cent. were paying-patients in first and second classes, the hospital having 600 beds. He defended the class system, as he would not deprive the rich man from having the luxuries which he could afford to pay for.

Professor GROBER

Briefly pointed out that fever departments were included in the German scheme because sometimes time was required to make sure of a diagnosis; and, secondly, because it was dangerous and undesirable to transfer a patient from one institution to another.

Sir HENRY BURDETT

Wished to explain that separate pavilions for pay patients must form part of any reorganised hospital system, but there was no difficulty about it whatever, as had been proved in the case of Sweden, Norway and Denmark, and Canada. He considered it would be madness to attempt a State system of hospitals in this country; the need was to correlate and gather up all that we had already; statesmen had agreed that along these lines a perfected system could be evolved; and as national insurance developed he believed a system would be put together which would stand. There should be throughout the country hospitals graduate classes under proper conditions, with the ablest men to demonstrate, so that the whole body of the medical profession might keep up to the highest standard, and every doctor working in a country district could have all the facilities which he needed.

The conclusion of a very full morning's debate was marked by a cordial vote of thanks being accorded by acclamation to the President, Dr. John Ryle, J.P.

